# First Reported Concomitant Pulsed-Field Ablation and Left Atrial Appendage Occlusion in a Patient With Prior Atrial Septal Defect Closure Device

**DOI:** 10.7759/cureus.114996

**Published:** 2026-08-22

**Authors:** Yu-Chiang Wang, Narut Prasitlumkum, Pattara Rattanawong

**Affiliations:** 1 Cardiovascular Medicine, University of Hawaiʻi at Mānoa, Honolulu, USA; 2 Cardiology, Pali Momi Medical Center, Honolulu, USA; 3 Cardiology, Hawaiʻi Pacific Health, Honolulu, USA

**Keywords:** atrial fibrillation, atrial septal defect closure, ct cardiac, intracardiac echocardiography, left atrial appendage occlusion, multimodality imaging, pulsed-field ablation, transesophageal echocardiography, transseptal puncture

## Abstract

Transseptal access in patients with prior atrial septal defect (ASD) closure devices is technically challenging, particularly when large-bore sheaths are required. We report a 59-year-old woman with longstanding persistent atrial fibrillation (AF) and prior ASD closure who underwent concomitant pulsed-field ablation (PFA) and left atrial appendage (LAA) occlusion. Preprocedural cardiac computed tomography identified a narrow (~5 mm) inferior native septal window. A multimodality imaging approach incorporating intracardiac echocardiography, transesophageal echocardiography, fluoroscopy, and CT integration enabled precise inferior transseptal puncture directed toward the LAA. This strategy allowed safe delivery of large sheaths and optimal coaxial alignment for Watchman implantation. Pulmonary vein isolation, posterior wall isolation, and cavotricuspid isthmus ablation were performed, followed by successful LAA occlusion without residual leak. To our knowledge, this is the first reported case of concomitant PFA and LAA occlusion performed through a single transseptal access in a patient with a prior ASD closure device. A focused multi-database literature search identified no previously reported case of this specific combination.

## Introduction

Atrial fibrillation (AF) frequently coexists with atrial septal defects (ASDs) and often persists after percutaneous ASD closure, particularly in older patients and those with longstanding atrial remodeling; consequently, some patients require rhythm-control and stroke-prevention procedures after their defect has already been closed with a device. Catheter ablation and left atrial appendage (LAA) occlusion are increasingly used to control the arrhythmia and to reduce thromboembolic risk, respectively, and both require transseptal access to the left atrium. Pulsed field ablation (PFA) is a nonthermal ablation modality that delivers high-voltage, microsecond electrical pulses to produce irreversible electroporation of cardiomyocytes; unlike thermal energy sources such as radiofrequency or cryoablation, it is relatively tissue-selective and tends to spare adjacent structures such as the esophagus and phrenic nerve. LAA occlusion is an alternative to long-term oral anticoagulation for stroke prevention in selected patients with nonvalvular AF who have an elevated thromboembolic risk together with a contraindication to, or intolerance of, long-term anticoagulation. In patients with a prior ASD closure device, transseptal puncture is technically challenging because of limited residual native septal tissue and the potential for device interaction, embolization, or cardiac perforation [1,2]. These challenges are compounded when large-bore sheaths are required, as with PFA and LAA occlusion. A single, well-positioned puncture through a narrow residual septal window must accommodate sequential large-caliber devices, leaving little margin for the trajectory adjustments available in a standard transseptal ablation. Although each element of this approach has been described individually - ablation, including PFA, in patients with septal occluder devices; concomitant PFA and LAA occlusion, including through a single transseptal access; and LAA occlusion in a patient with a prior ASD device - their integration in a single patient has not been reported. We report what is, to our knowledge, the first concomitant PFA and LAA occlusion performed through a single transseptal access in a patient with a prior ASD closure device, enabled by a multimodality imaging strategy; the literature search supporting this novelty claim is summarized in the discussion.

## Case presentation

A 59-year-old woman with a history of antineutrophil cytoplasmic antibodies (ANCA)-associated vasculitis, obstructive sleep apnea, hypertension, and longstanding persistent atrial fibrillation presented with progressive fatigue and exertional dyspnea. She had previously undergone ASD closure with a 13-mm Amplatzer device (Abbott, Plymouth, MN, USA) eight months ago. Ambulatory monitoring demonstrated persistent AF. Her CHA₂DS₂-VASc score was two, indicating an elevated risk of thromboembolism; however, she declined long-term oral anticoagulation because of easy bruising and a history of chronic hematuria. Given her symptomatic arrhythmia and intolerance to anticoagulation, a combined strategy of AF ablation and LAA occlusion was pursued.

**Figure 1:**
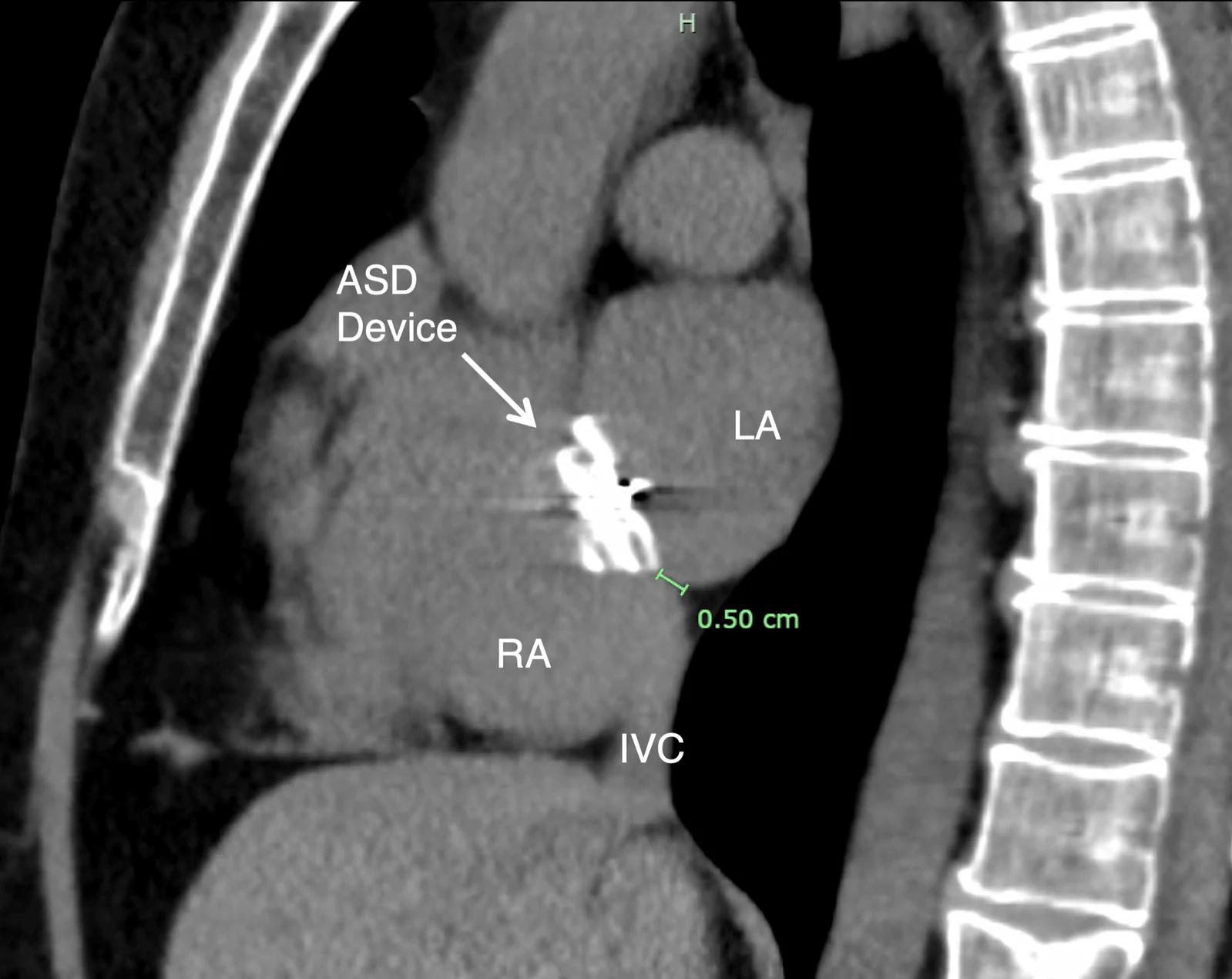
Preprocedural imaging - cardiac computed tomography Cardiac computed tomography demonstrating a limited (~5 mm) native septal tissue window inferior to the atrial septal defect (ASD) closure device. ASD: atrial septal defect; IVC: inferior vena cava; LA: left atrium; LAA: left atrial appendage; RA: right atrium.

Preprocedural cardiac computed tomography demonstrated a limited native septal tissue window measuring approximately 5 mm inferior to the ASD closure device (Figure 1), which was considered suitable for transseptal access with careful technique and imaging guidance.

**Figure 2:**
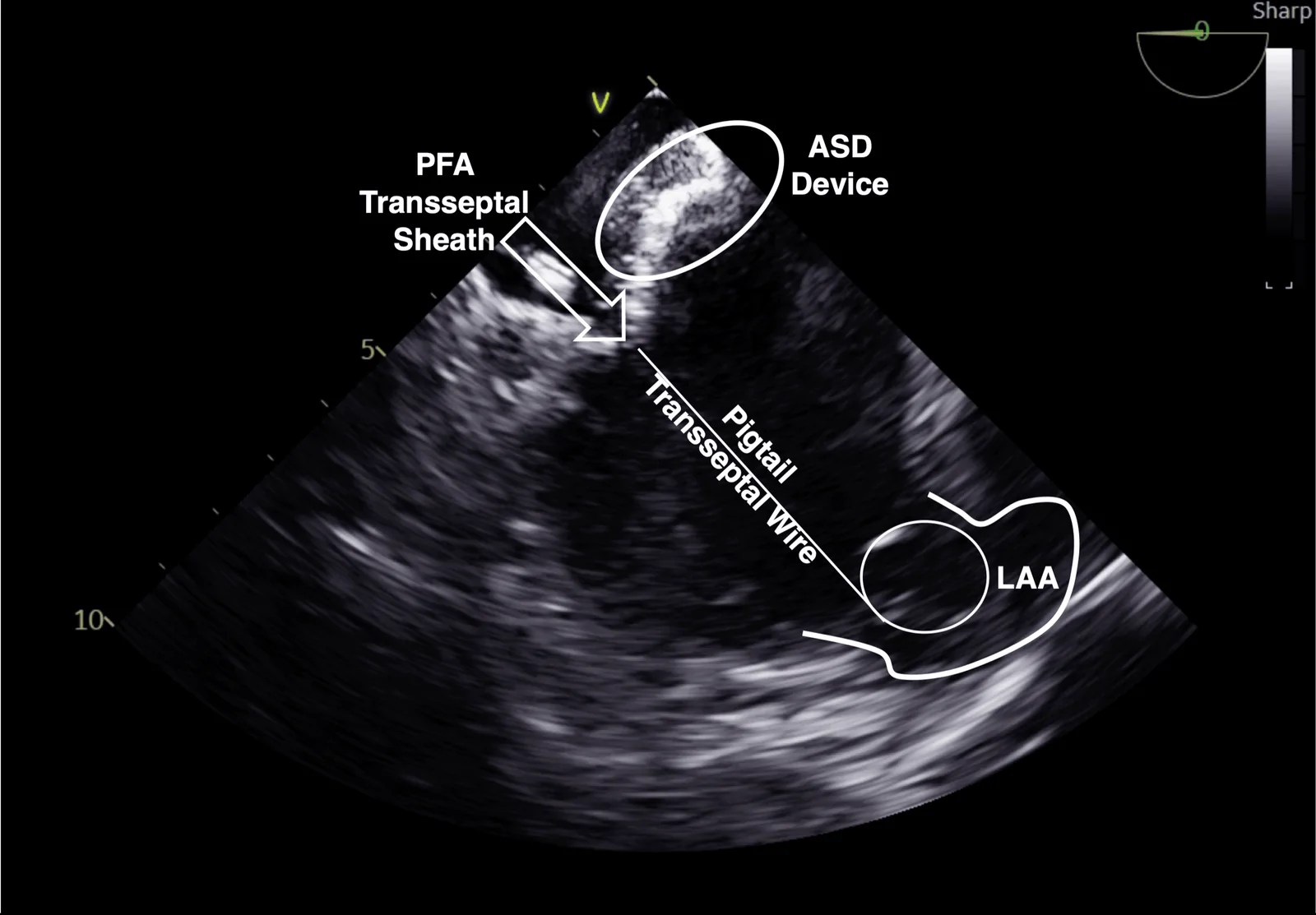
Pre-transseptal puncture intracardiac echocardiography Pre-transseptal puncture intracardiac echocardiography demonstrating an inferior transseptal trajectory directed toward the left atrial appendage, inferior to the ASD closure device. ASD: atrial septal defect; PFA: pulsed field ablation; LA: left atrium; LAA: left atrial appendage.

The procedure was performed under general anesthesia with continuous electrocardiographic and hemodynamic monitoring. A multimodality imaging strategy incorporating intracardiac echocardiography (Figure 2), transesophageal echocardiography (Video 1), fluoroscopy (Video 2), and CT integration was used to guide transseptal access and catheter positioning. The pre-existing ASD closure device was well-seated with no baseline residual interatrial shunt on pre-procedural imaging. A complete list of equipment is provided in Table 1. 

**Video 1:** Transesophageal echocardiography (TEE) (A) TEE at 45° confirmed that the needle tip was positioned just inferior to the ASD closure device. (B) After puncture, TEE at 90° demonstrated the transseptal wire crossing the septum inferior to the device, within the native tissue plane. ASD: atrial septal defect; TEE: transesophageal echocardiography.

**Video 2:** Fluoroscopic-guided inferior transseptal puncture Fluoroscopic-guided inferior transseptal puncture demonstrating safe traversal of the septum inferior to the atrial septal defect (ASD) closure device.

**Table 1:** Equipment used for the concomitant pulsed-field ablation and left atrial appendage occlusion procedure. Equipment used for the concomitant pulsed-field ablation and left atrial appendage occlusion procedure. AF, atrial fibrillation; AFL, atrial flutter; CS, coronary sinus; CTI, cavotricuspid isthmus; ICE, intracardiac echocardiography; LA, left atrium; LAA, left atrial appendage; PFA, pulsed-field ablation; TEE, transesophageal echocardiography. Manufacturers: CARTO 3 system, coronary sinus catheter, Optrell mapping catheter, and NuVision intracardiac echocardiography catheter (Biosense Webster, Inc., Irvine, CA, USA); Farawave/Farapulse ablation catheter, Faradrive steerable sheath, VersaCross Connect steerable sheath, VersaCross transseptal (radiofrequency) pigtail wire, and Watchman FLX (Boston Scientific, Marlborough, MA, USA); Perclose ProGlide (Abbott, Abbott Park, IL, USA).

Equipment List		
Concomitant PFA AF/AFL ablation & Watchman implantation		
Category	Equipment	Details
Mapping system	Biosense Webster CARTO 3	3D electroanatomic mapping
Catheters	Biosense CS catheter	CS recording/pacing
	Biosense Optrell	Mapping catheter
	Farawave (Farapulse)	PFA ablation, 31 mm
	Biosense NuVision ICE	Intracardiac echo
Sheaths	Farapulse Faradrive steerable sheath	16 Fr
	Boston Scientific VersaCross Connect steerable sheath	17 Fr
Watchman device	Boston Scientific Watchman FLX	31 mm
Transseptal access	VersaCross pigtail wire + RF energy	Transseptal puncture
Hemostasis	Perclose ProGlide	Double pre-close technique
Imaging	TEE, Biosense NuVision ICE, cardiac CT	Watchman sizing & deployment

Transseptal puncture was intentionally directed inferior to the ASD closure device, targeting the residual native septal tissue plane between the device and the septal free wall. The residual native septum was relatively fibrotic and noticeably firmer than a typical fossa ovalis, requiring slightly greater tactile force to achieve adequate tenting. Given the limited septal tissue and proximity of the ASD closure device, forward pressure was applied gently and incrementally under continuous imaging guidance. RF-assisted puncture with the VersaCross system (Boston Scientific, Marlborough, MA, USA) subsequently allowed controlled traversal without device interaction or displacement. The trajectory was deliberately oriented toward the left atrial appendage rather than the pulmonary veins to optimize subsequent device delivery. Under intracardiac echocardiographic and transesophageal echocardiographic guidance, a VersaCross system was used to achieve transseptal access, with imaging confirming safe crossing of the septum inferior to the device without interaction or displacement.

**Figure 3:**
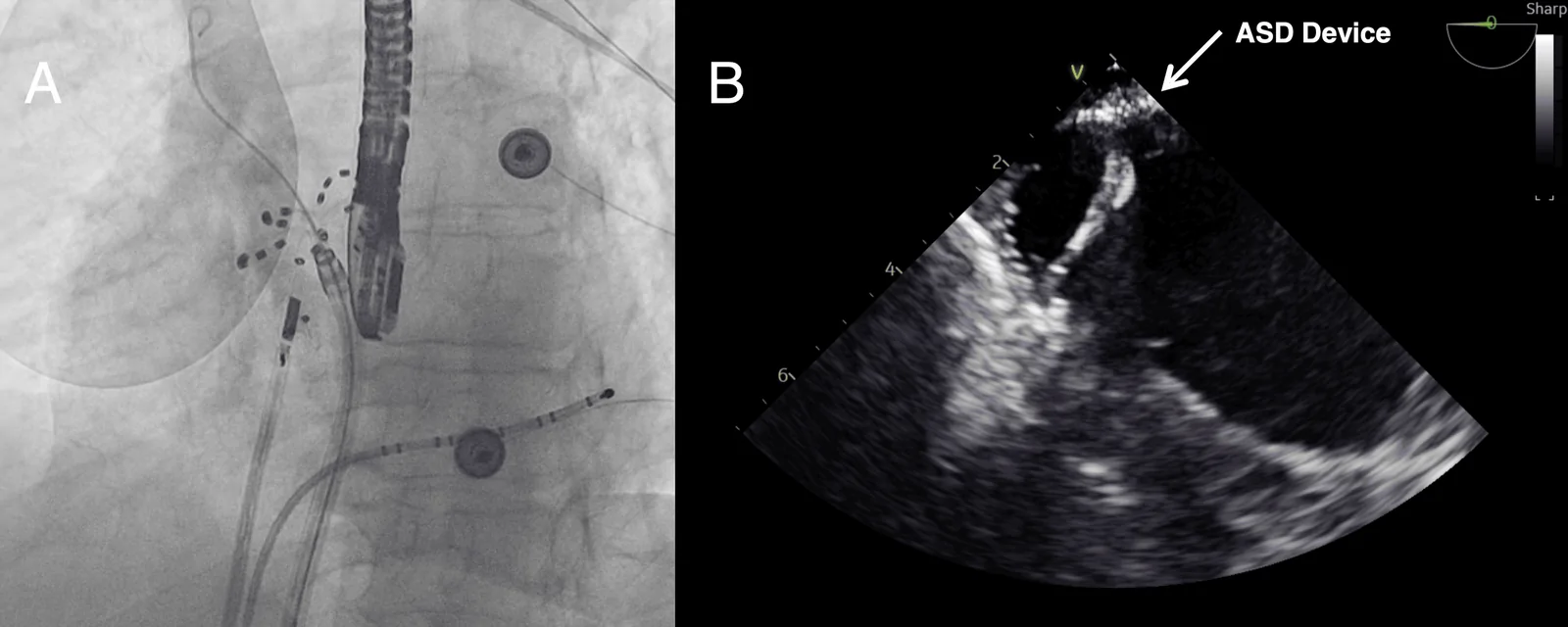
Procedural imaging (A) Fluoroscopy and (B) intracardiac echocardiography confirming the position of the Farawave ablation catheter, with care taken to avoid contact between the catheter spines and the atrial septal defect (ASD) closure device.

Following left atrial access, PFA was performed using a Farawave catheter (Boston Scientific) (Figure 3A, 3B). 

**Figure 4:**
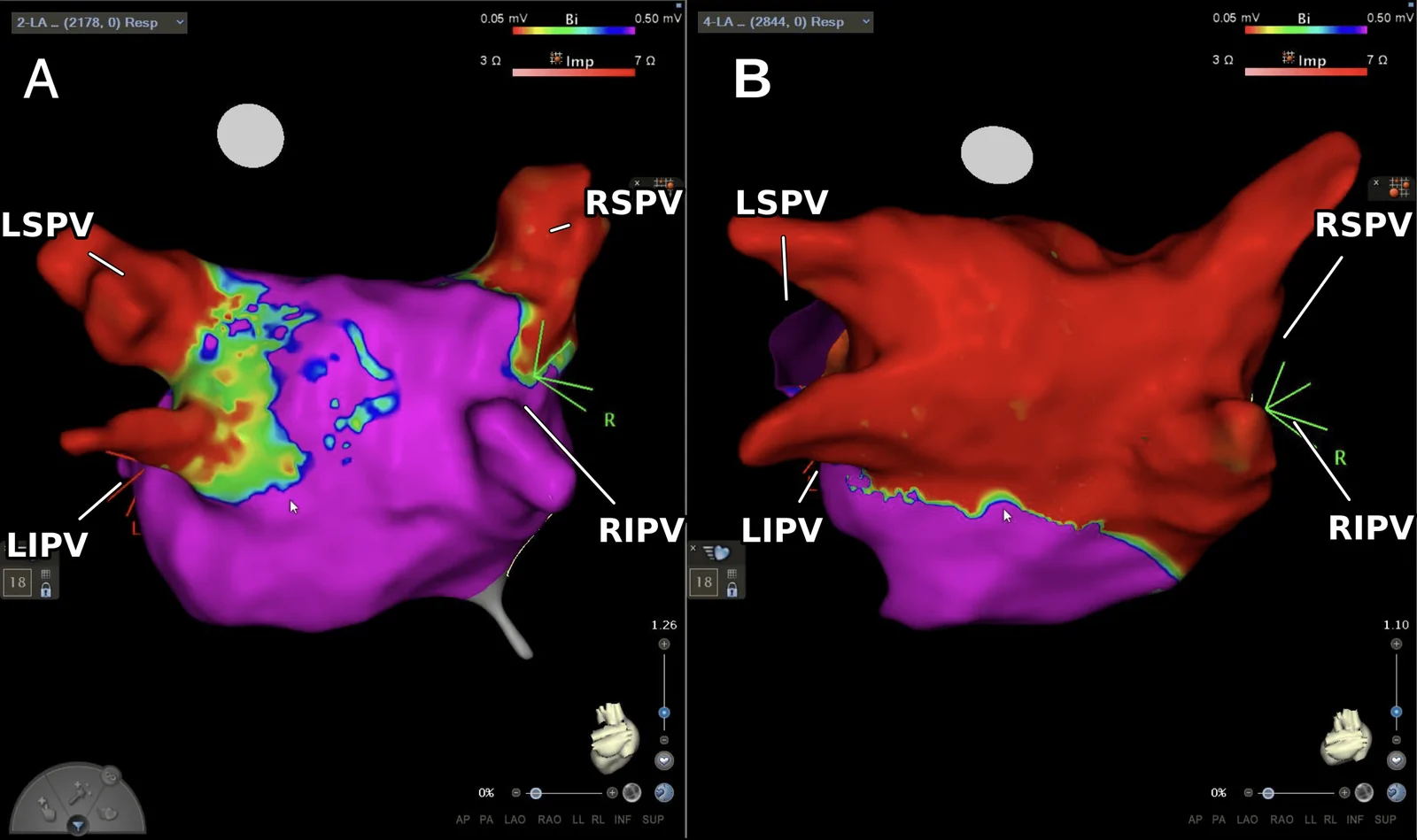
Procedural electroanatomic mapping. Procedural electroanatomic mapping. (A) Pre-ablation and (B) post-ablation left atrial bipolar voltage maps (posteroanterior projection) displayed on a color scale of 0.05 – 0.5 mV (red, low voltage/scar; purple, preserved voltage), with the pulmonary-vein ostia labeled; Optrell mapping was used to avoid spline entrapment within the ASD closure device. Mapping was performed in sinus rhythm. Post-ablation mapping confirmed acute wide antral pulmonary vein isolation with posterior wall isolation, defined by entrance and exit block with elimination of pulmonary-vein potentials. ASD: atrial septal defect; LSPV/LIPV: left superior/inferior pulmonary veins; RSPV/RIPV: right superior/inferior pulmonary veins.

Wide antral pulmonary vein isolation was achieved, followed by posterior left atrial wall isolation with creation of roof and floor lines to address the substrate of longstanding persistent AF. During the procedure, typical atrial flutter was induced and successfully treated with cavotricuspid isthmus ablation using PFA energy, resulting in bidirectional conduction block. Post-ablation electroanatomic mapping confirmed durable isolation (Figure 4A, 4B).

After completion of ablation, the transseptal sheath was exchanged for a 17-Fr VersaCross Connect steerable sheath to allow access for the Watchman delivery system. The inferiorly directed transseptal trajectory provided excellent coaxial alignment with the LAA, facilitating device delivery. A 31-mm Watchman device (Boston Scientific) was deployed successfully with appropriate compression and stable positioning confirmed by tug testing. Transesophageal echocardiography (Video 3) and angiography (Video 4) demonstrated no residual peri-device leak.

**Video 3:** Transesophageal echocardiography after Watchman deployment. Post-deployment transesophageal echocardiography demonstrated optimal Watchman positioning without residual leak at (A) 0°, (B) 45°, (C) 90°, and (D) 120°.

**Video 4:** Fluoroscopic angiography after Watchman deployment. Fluoroscopic angiography demonstrating optimal Watchman device positioning without residual leak.

There were no immediate procedural complications. A small inferior iatrogenic patent foramen ovale was observed following transseptal access without hemodynamic significance (Video 5). Follow-up TEE after 45 days of the procedure demonstrated only a trivial left-to-right interatrial shunt at the transseptal puncture site without hemodynamic significance; therefore, no additional intervention was required. At three-month follow-up, the patient remained asymptomatic and in sinus rhythm without recurrence of AF.

**Video 5:** Post-procedural three-dimensional transesophageal echocardiography (3D TEE) (A) 3D TEE after Watchman deployment demonstrated optimal positioning and absence of peridevice leak. (B) 3D TEE showing a well-seated atrial septal defect (ASD) closure device and a small inferior iatrogenic ASD.

The patient tolerated the procedure well and was discharged after overnight observation. She was treated with short-term anticoagulation followed by dual antiplatelet therapy according to standard post-LAA occlusion protocols. Following Watchman implantation, the patient received apixaban 5 mg twice daily and aspirin 81 mg daily for 45 days. Surveillance TEE performed after 45 days demonstrated a well-seated Watchman device without significant peri-device leak or device-related thrombus. Apixaban was subsequently discontinued, and aspirin 81 mg daily and clopidogrel 75 mg daily were continued for six months, followed by aspirin 81 mg daily indefinitely.

## Discussion

Concomitant PFA and LAA occlusion is an emerging one-stop strategy that has recently been shown to be feasible and safe, including through a single transseptal access [3,4]. Ablation, including PFA, has likewise been performed in patients with prior septal occluder devices [1,2,5-9], and LAA occlusion has been performed in such a patient [10]. To our knowledge, however, the combination of these approaches - concomitant PFA and LAA occlusion through a single transseptal access in a patient with a prior ASD closure device, in whom limited residual native septum and the large-bore sheaths required for PFA and LAA occlusion compound the transseptal challenge - has not previously been reported. Treatment options were discussed through shared decision-making, including continued medical therapy with oral anticoagulation, staged AF ablation and LAA occlusion, and surgical or epicardial LAA exclusion. The patient declined long-term anticoagulation because of recurrent bruising and chronic hematuria. A staged approach would also have required repeat transseptal access through the limited residual native septum, while surgical or epicardial approaches were considered more invasive. The patient therefore elected concomitant percutaneous AF ablation and LAA occlusion.

**Figure 5:**
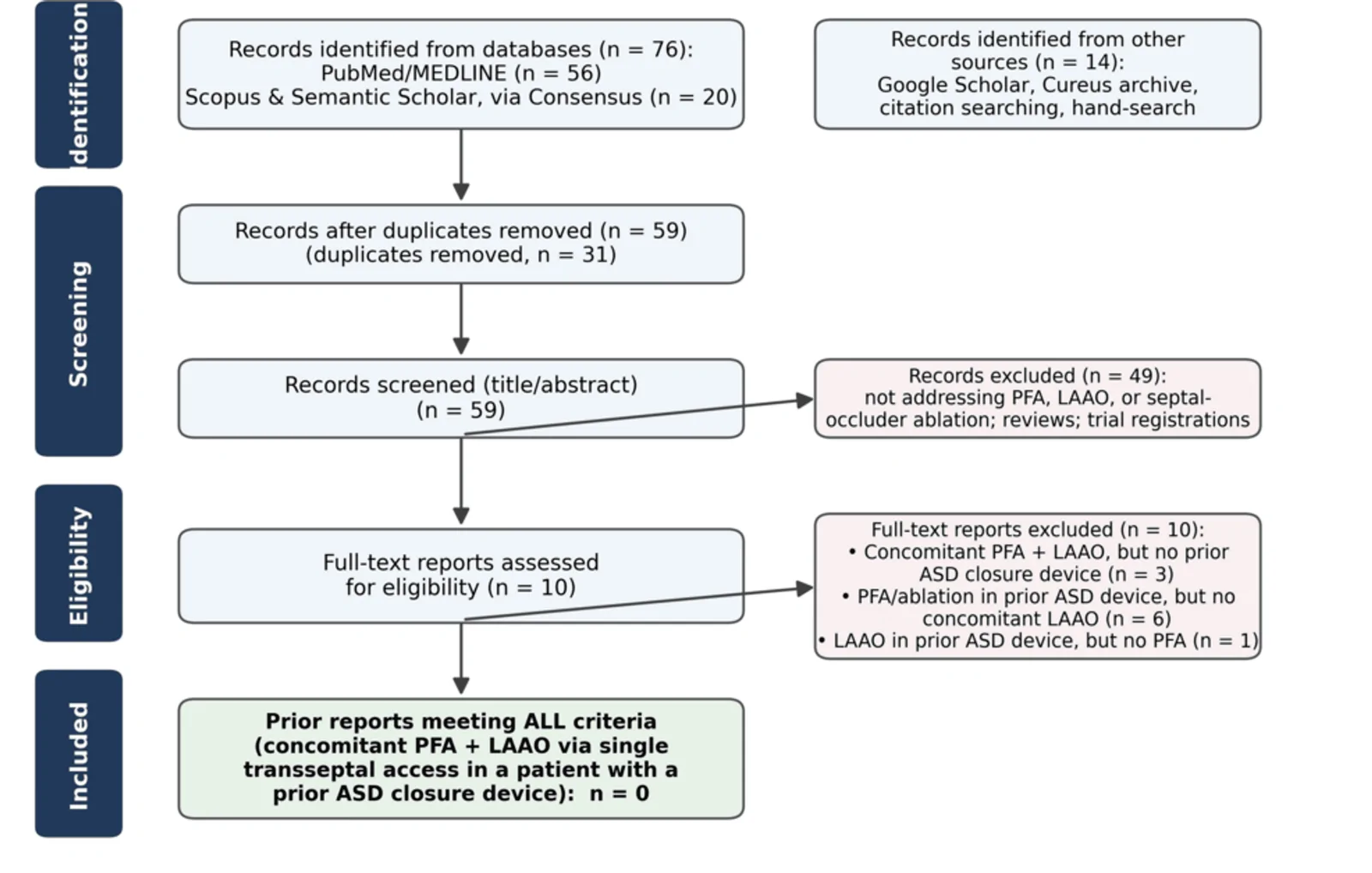
Literature-search flow diagram (PRISMA 2020-style) for the novelty assessment. Records were identified from PubMed/MEDLINE, Scopus and Semantic Scholar (via Consensus), Google Scholar, and the Cureus archive, supplemented by citation searching, through July 2026. After de-duplication, screening, and full-text assessment, no prior report described the specific combination of concomitant PFA and LAAO through a single transseptal access in a patient with a prior ASD closure device. As a focused (non-systematic) search limited to selected databases and English-language, indexed sources, our novelty assessment is subject to possible search and database bias. ASD: atrial septal defect; LAAO: left atrial appendage occlusion; PFA: pulsed-field ablation; PRISMA: Preferred Reporting Items for Systematic Reviews and Meta-Analyses [11]

To substantiate this, we performed a focused literature search of PubMed/MEDLINE, Scopus and Semantic Scholar (via Consensus), Google Scholar, and the Cureus archive, supplemented by citation searching, from database inception through July 2026 (Appendix 1), combining the terms pulsed field ablation, left atrial appendage occlusion or closure, Watchman, transseptal puncture, and atrial septal defect closure device or septal occluder (full search strings, Appendix 2). Eligible reports had to describe concomitant PFA and LAA occlusion through a single transseptal access in a patient with a prior ASD closure device (eligibility criteria, Appendix 3). After removal of duplicates, 59 records were screened and 10 full-text reports were assessed; none met all four criteria (Figure 5; the assessed reports and reasons for exclusion are listed in Appendix 4).

Transseptal access in patients with ASD closure devices is inherently challenging due to limited available septal tissue and increased risk of device interaction, embolization, or cardiac perforation [5,12]. In this case, preprocedural computed tomography was critical in identifying a narrow but usable inferior septal window. This finding allowed for precise procedural planning and safe execution.

Multimodality imaging played a central role in procedural success [10]. The integration of computed tomography, intracardiac echocardiography, transesophageal echocardiography, and fluoroscopy enabled accurate localization of the residual septal tissue, real-time visualization of the ASD closure device, and confirmation of safe transseptal traversal. This approach is particularly important when large-bore sheaths are required, as in AF PFA and LAA occlusion.

Equally important was the deliberate orientation of the transseptal trajectory toward the LAA. This strategy provided optimal coaxial alignment for device delivery and eliminated the need for repeat transseptal puncture. In patients with limited septal access, achieving the correct trajectory during the initial puncture is critical for procedural safety and efficiency.

The combined strategy of AF ablation and LAA occlusion in a single session offers several advantages, including avoidance of repeat transseptal access and reduction of procedural burden [13]. This approach is particularly advantageous in anatomically complex cases such as this one.

Finally, PFA represents a promising nonthermal modality that may reduce collateral tissue injury and is well-suited for complex anatomical scenarios [14]. The choice of PFA rather than radiofrequency ablation was particularly relevant in this anatomically constrained case. Because the ASD closure device occupied most of the interatrial septum and was adjacent to the transseptal working field, minimizing catheter manipulation and procedural complexity was desirable. PFA enabled rapid pulmonary vein and posterior wall ablation without extensive point-by-point thermal lesion delivery, thereby limiting repeated manipulation across the narrow residual septal window. Throughout the procedure, catheter positioning was carefully monitored to avoid direct interaction between the PFA catheter and the ASD closure device. This strategy also facilitated preservation of a single transseptal access for subsequent LAA occlusion.

This case highlights several practical lessons: strategies for transseptal access in patients with a prior ASD closure device, the central role of multimodality imaging in guiding complex transseptal puncture, and the feasibility of a combined pulsed-field ablation and left atrial appendage occlusion approach in appropriately selected patients.

## Conclusions

This case illustrates the potential feasibility of a single-procedure strategy combining PFA and LAA occlusion in a carefully selected patient with a prior ASD closure device, enabled by precise multimodality imaging and careful transseptal planning. Careful preprocedural computed tomography and intraprocedural multimodality imaging facilitated identification of a usable septal window and safe transseptal access in this case when a septal occluder device was present. This report has several limitations. It describes a single patient at a single center with favorable anatomy-a discrete, usable inferior septal window-so the findings may not generalize to patients with narrower residual septa or with different closure devices (e.g., Amplatzer Cribriform or Gore Cardioform). There was no comparator, the approach is resource-intensive (requiring CT, intracardiac and transesophageal echocardiography, and fluoroscopy), and follow-up was limited to the short term; comparative safety, efficacy, and durability therefore cannot be established. As a focused rather than exhaustive search, the novelty assessment is also subject to possible search and database bias.

## Appendices

Appendix 1: Databases, coverage, and dates

A focused (non-systematic) search was performed to establish whether the specific four-component combination had been reported previously. Databases and sources were searched from inception through 31 July 2026, without language restriction:

PubMed/MEDLINE (NLM)

Scopus and Semantic Scholar, queried via Consensus

Google Scholar (first 100 results screened)

Cureus journal archive

Backward/forward citation searching of the reports listed below

Appendix 2: Exact query strings

PubMed/MEDLINE (searched 31 July 2026)

(("pulsed field ablation"[tiab] OR "pulsed-field ablation"[tiab] OR PFA[tiab])

 AND ("left atrial appendage"[tiab] AND (occlusion[tiab] OR closure[tiab] OR Watchman[tiab] OR LAAO[tiab]))) OR (("atrial septal defect"[tiab] OR "septal occluder"[tiab] OR "septal closure device"[tiab]) AND (ablation[tiab] OR transseptal[tiab]) AND ("atrial fibrillation"[tiab] OR "left atrial appendage"[tiab]))

Scopus and Semantic Scholar, via Consensus (searched July 2026)

"concomitant pulsed field ablation and left atrial appendage occlusion through a single transseptal access in a patient with a prior atrial septal defect closure device"

"transseptal puncture for atrial fibrillation ablation or left atrial appendage occlusion in patients with an atrial septal defect closure device / septal occluder"

Google Scholar (searched July 2026; first 100 results screened)

("pulsed field ablation" OR PFA) ("left atrial appendage" occlusion OR closure OR Watchman) ("atrial septal defect" closure device OR "septal occluder") transseptal

Cureus archive (searched July 2026)

pulsed field ablation left atrial appendage occlusion atrial septal defect closure device

Appendix 3: Eligibility criterion

An eligible prior report had to describe all four of:

pulsed-field ablation (PFA);

left atrial appendage (LAA) occlusion;

performed concomitantly through a single transseptal access; and

in a patient with a prior atrial septal defect (ASD) closure device.

Appendix 4: Full-text reports assessed, with reasons for exclusion

Ten full-text reports were assessed. Each addressed some, but not all four, of the eligibility criteria; none met all four, establishing that the specific combination had not been previously reported (Figure 5). The exclusion table is provided as a separate file and reproduced below (Table 2).

**Table 2:** Full-text reports assessed in the novelty search, with reasons for exclusion Search dates: database inception through 31 July 2026. Eligibility required all four of: (i) pulsed-field ablation (PFA); (ii) left atrial appendage (LAA) occlusion; (iii) concomitant, through a single transseptal access; (iv) in a patient with a prior atrial septal defect (ASD) closure device. Ten full-text reports were assessed; none met all four criteria (0 eligible).

No.	Match category	Report (citation)	DOI	Reason not eligible
1	Concomitant PFA + LAAO, no prior ASD device	Tabaja C, Mdaihly M, Besir B, et al. [3] Combined pulsed field ablation of atrial fibrillation and left atrial appendage occlusion: a single-center experience. Heart Rhythm. 2025.	10.1016/j.hrthm.2025.12.017	No prior ASD closure device
2	Concomitant PFA + LAAO, no prior ASD device	Heeger CH, Rolfes H, Böttcher L, et al. [4] Streamlined concomitant pulsed field ablation–based pulmonary vein isolation and left atrial appendage occlusion via a single venous access approach: a case report. Eur Heart J Case Rep. 2025.	10.1093/ehjcr/ytaf350	No prior ASD closure device
3	Concomitant PFA + LAAO, no prior ASD device	Schiavone M, et al. [15] Pulsed field ablation with a pentaspline catheter and concomitant left atrial appendage closure for long-standing persistent atrial fibrillation: comparison with a radiofrequency cohort. Heart Rhythm. 2026.	10.1016/j.hrthm.2026.04.018	No prior ASD closure device
4	Ablation in prior ASD/occluder device, no LAAO	Santangeli P, Di Biase L, Burkhardt JD, et al. [1] Transseptal access and atrial fibrillation ablation guided by intracardiac echocardiography in patients with atrial septal closure devices. Heart Rhythm. 2011.	10.1016/j.hrthm.2011.06.023	No concomitant LAA occlusion
5	Ablation in prior ASD/occluder device, no LAAO	Li X, Wissner E, Kamioka M, et al. [6] Safety and feasibility of transseptal puncture for atrial fibrillation ablation in patients with atrial septal defect closure devices. Heart Rhythm. 2014.	10.1016/j.hrthm.2013.11.011	No concomitant LAA occlusion
6	Ablation in prior ASD/occluder device, no LAAO	Guo Q, Sang C, Bai R, et al. [5] Transseptal puncture in patients with septal occluder devices during catheter ablation of atrial fibrillation. EuroIntervention. 2022.	10.4244/EIJ-D-21-00340	No concomitant LAA occlusion
7	Ablation in prior ASD/occluder device, no LAAO	Oliveira M, Sousa L, Trindade A, et al. [8] Single puncture approach guided by transesophageal echocardiography for atrial fibrillation ablation in a patient with prior percutaneous septal closure: case report. Eur Heart J Case Rep. 2023.	10.1093/ehjcr/ytad139	No concomitant LAA occlusion
8	Ablation in prior ASD/occluder device, no LAAO	Hu J, Ding L, Gunawan E, et al. [9] Successful treatment of atrial flutter post-radiofrequency ablation for atrial fibrillation following atrial septal defect occlusion: a case report of pulsed field ablation. Eur Heart J Case Rep. 2024.	10.1093/ehjcr/ytae558	No concomitant LAA occlusion
9	Ablation in prior ASD/occluder device, no LAAO	Nyuta E, Takemoto M, Yoshida D, et al. [7] Direct transseptal puncture across an atrial septal defect closure device for ablation of atrial fibrillation. HeartRhythm Case Rep. 2025.	10.1016/j.hrcr.2025.05.014	No concomitant LAA occlusion
10	LAAO in prior ASD device, no PFA	Korsholm K, Jensen JM, Nielsen-Kudsk JE. [10] Left atrial appendage occlusion guided by intracardiac echocardiography in a patient with a 34 mm atrial septal defect occluder: a case report. Eur Heart J Case Rep. 2023.	10.1093/ehjcr/ytad571	No pulsed-field ablation
